# Thyroid heterotopia revealed by a cervical subcutaneous nodule: a clinical case report

**DOI:** 10.11604/pamj.2021.40.60.27683

**Published:** 2021-09-27

**Authors:** Yannick Mossus, David Mindja Eko, Leonel Atanga, Roger Christian Meva’a Biouele, Adèle-Rose Ngo Nyeki, François Djomou, Alexis Ndjolo

**Affiliations:** 1 Department of Ophthalmology, Ear, Nose and Throat (ENT) and Stomatology, Faculty of Medicine and Biomedical Sciences, University of Yaoundé I, Yaoundé, Cameroon

**Keywords:** Thyroid heterotopia, neck, clinical, case report

## Abstract

Thyroid heterotopia is an abnormal localization of normal thyroid tissue coexisting with a normal organ on a normal localization. It is a rare condition with a frequency that is not well known in the literature. We report a case of thyroid heterotopia in a 30 month-old girl referred for a painless lower antero-cervical nodule that has been developing for one year with past history no contributory. The clinical examination found a subcutaneous formation mobile in relation to the different deep and superficial planes with bilateral angulomandibular micro-lymphadenopathy. Ultrasounds of the soft parts of the neck showed hypoechogenic tissue reminiscent of thyroid tissue, the thyroid was in place. The thyroid hormone profile was normal. In the absence of a functional scintigraphy device, the diagnosis was confirmed with a pathology exam after surgery. We discuss the diagnostic relevance of thyroid heterotopia in front of any anterior nodular formation of the neck.

## Introduction

Thyroid heterotopia is an abnormal localization of normal thyroid tissue coexisting with a normal organ on a normal localization. It is a congenital anomaly due to a defect in maturation and migration of thyroid tissues [1,2]. It is a rare condition whose frequency is not well known in the general literature. However, it is one of the thyroid ectopias, the prevalence of which varies from 1/4000 to 1/8000 cases of hypothyroidism with a female predominance (sex ratio 1/4) [3,4]. Ectopic localizations of the thyroid gland occur along the thyroglossal tract with a predominance in 90% of cases on the base of the tongue [3,4]. However, a few cases of extra-cervical, mediastinal or pre-sternal localizations have been reported in the literature [1,2,5].

## Patient and observation

**Patient information:** it is about a girl of 2 years 6 months not yet schooled brought to consult for cervical nodule. No particularities in pregnancy and childbirth were noted. Since birth, the child has never presented an episode of illness requiring hospitalization and the vaccination follow-up was satisfactory. The family history was not contributory and there was not familial goiter.

**Figure 1:**
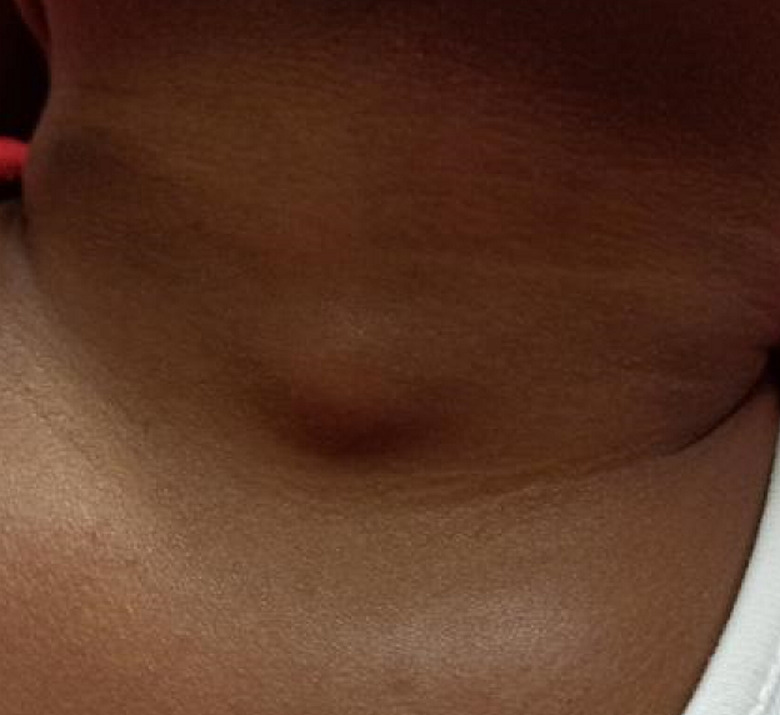
clinical presentation of the nodule

**Figure 2:**
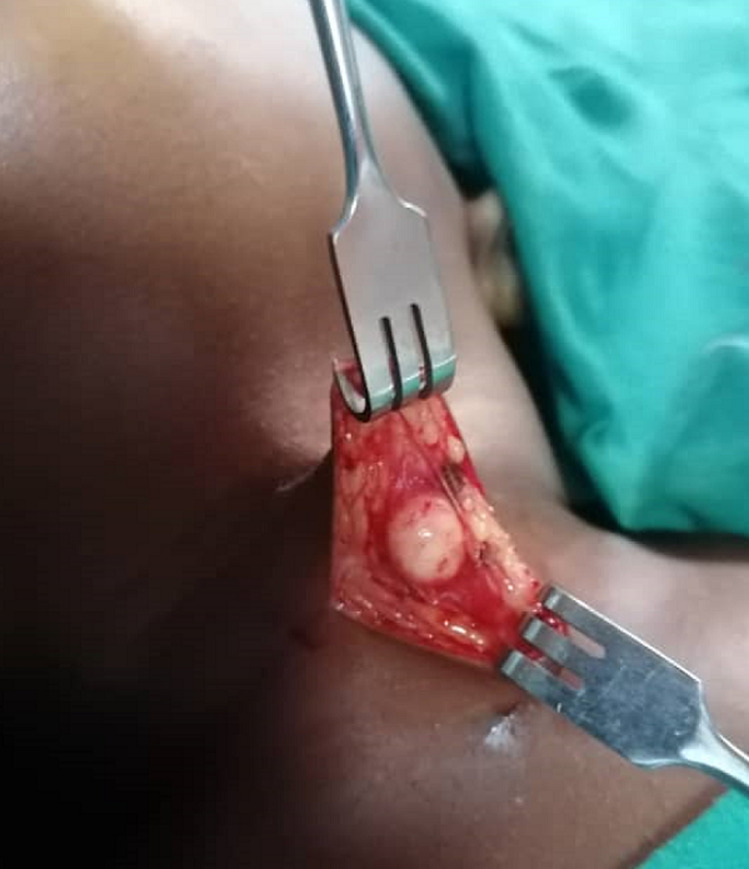
intraoperative view of the nodule after neck excision

**Clinical findings:** on clinical examination, a painless and well-defined nodule was found with a 10 mm diameter opposite the thyroid compartment, with elastic consistency, mobile with respect to the different superficial and deep planes and the skin opposite was healthy (Figure 1, Figure 2). The nodule did not ascend with swallowing. Examination of the different lymph node areas revealed bilateral and infra-centimetric subangulo-mandibular lymphadenopathy.

**Timeline of current episode:** one-year ago before the consultation, parents observed a nodule on the child neck and no related consultation or care has been administered to the child. July 2020, patient has been consulted and diagnostic assessments were performed in the same time. December 2020, surgery and histopathological analysis of the specimen were made.

**Figure 3:**
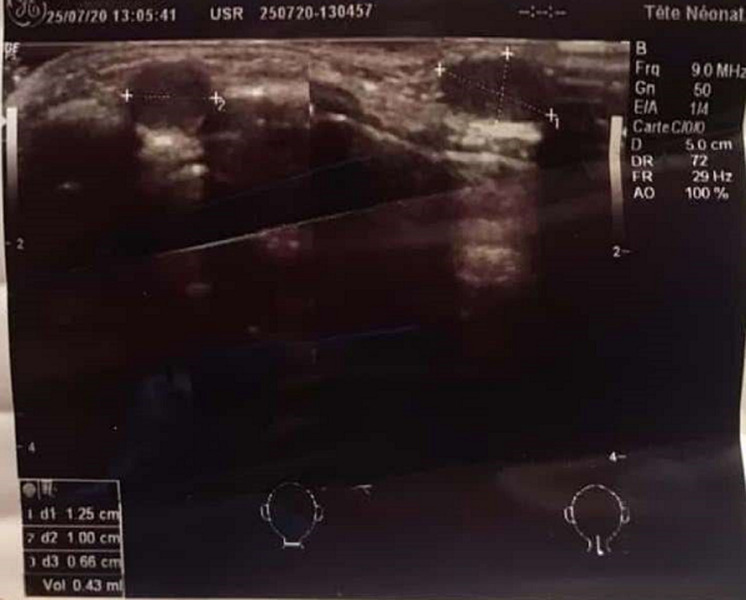
ultrasound of soft tissues of the patient neck

**Diagnostic assessment:** the ultrasound performed found a solid median nodule opposite the trachea 15 x 10 x 6 mm subcutaneous with an echo-structure similar to that of the normal thyroid which was in normal place (Figure 3). That also noted bilateral jugulocarotid lymphadenopathy. The thyroid hormone test based on thyroid stimulating hormone (TSH) was normal.

**Diagnosis:** the assessment was compatible with a thyroid heterotopia in subhyoid site. Other diagnoses considered were cyst of the thyroglossal tract or a medial cyst of the neck.

**Therapeutic interventions:** the surgical excision was performed under general anesthesia with orotracheal intubation (Figure 2). Surgical findings were a nodule without relationship with the underlying thyroid compartment or the hyoid bone.

**Figure 4:**
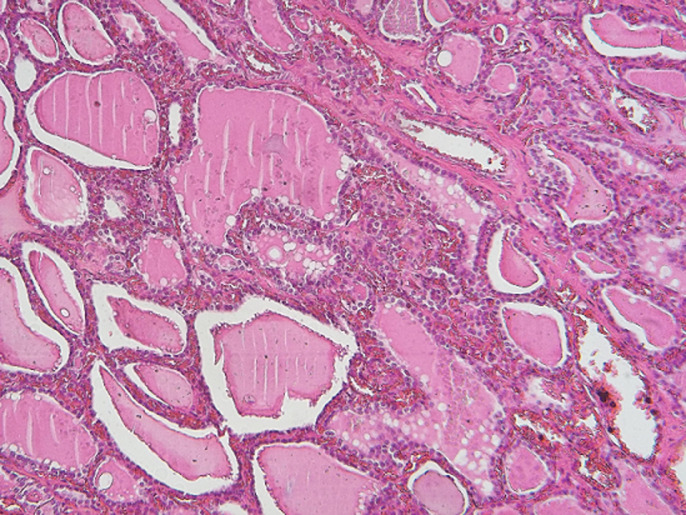
pathological analysis of the surgical specimen

**Follow-up and outcome of interventions:** the immediate and early post-operative consequences after two days of hospitalization were simple. Pathological analysis confirmed the thyroid nature of the operative specimen (Figure 4). One month after surgery, hormone balance was still normal. Three months after the surgery, there was not unsightly scar on the patient´s neck.

**Patient perspective:** the parents were satisfied with the treatment and expect that the nodule will not recur. In that perspective they ensure to continue clinical follow-up in each 6-months for two years.

**Informed consent:** the consent form has been applied before the surgery and mentioned the possibility to use photos and de-identified information, in the aim to share our experience. The parents signed the form after fully explanations.

## Discussion

Congenital thyroid abnormalities and variants of normal relate to size, shape, location or vascularity. Heterotopias can be discovered at any age of life since they are of embryonic origin. The age of discovery of ectopic thyroids varies from 6 to 50 years according to the literature [3,6,7]. Due to a quiet symptomatology and a thyroid normally in place, thyroid heterotopias are later discovered. The early diagnosis in our case could be explained by the superficial location of the nodule. The youngest subject observed in the literature was six and a half years old [3]. The subject observed is female as found by the vast majority of authors with a sex ratio ranging from 1/2 to 1/4 [3,4,6].

Thyroid heterotopias can sit along the embryonic migration path of the thyroid [2]. The most frequently encountered site is the base of the tongue (90%), and sub-hyoid localizations are the rarest [2]. A few cases of extra-cervical heterotopias have been reported in the literature [1,2,5]. The hormonal balance in heterotopias remains normal due to a normally located and functional gland, unlike ectopias which are accompanied by varying degrees of hypothyroidism [7]. The observed patient had a normal assessment, explained by the coexistence of a functional thyroid in a normal position. Mazouz *et al*.in 2016 reported a case of mediastinal heterotopia accompanied by thyrotoxicosis [2].

The sensitivity of ultrasound in the diagnosis of masses and nodules of the soft parts of the neck is high, so it remains the first-line examination. The diagnosis of secondary locations of normal or abnormal thyroid tissue is made by scintigraphy [8]. The technetium 99 m scintigraphy or better with iodine 124 makes it possible to demonstrate hyperfixation outside the normal thyroid compartment. The latter remains the method of choice in terms of sensitivity and specificity [9]. In our case, the diagnosis was suggested by cervical ultrasound with ectopic tissue with ultrasound characteristics identical to the thyroid tissue. Given the current unavailability of a scintigraphy device and the unsightly presentation of the nodule, we did a pathological analysis on the surgical specimen to confirm the diagnosis.

Although the literature is not unanimous on the treatment of cases of thyroid heterotopia, some authors have recommended a wait-and-see attitude towards pediatric subjects with these conditions [10]. Hormone therapy is indicated in cases of pure ectopia associated with hypothyroidism [11]. Surgery is an alternative recommended by some authors when the ectopic tissue becomes tumoral [1,2,5]. We performed the surgical removal of the nodule for various reasons. The nodule was subcutaneous and accessible, the ultrasounds had found a normal thyroid in a normal situation, the thyroid hormone balance was normal and finally the actual absence of scintigraphy that could help in the diagnosis of the tissue nature.

## Conclusion

Thyroid heterotopia is a rare condition. It should be considered in front of any anterior and midline neck formation without thyroid function disorder. Iodine scintigraphy remains the gold standard for thyroid heterotopia, but given its unavailability in our practice context, diagnostic confirmation is made after anatomopathological analysis of the surgical specimen.
